# Ribosome biogenesis in disease: new players and therapeutic targets

**DOI:** 10.1038/s41392-022-01285-4

**Published:** 2023-01-09

**Authors:** Lijuan Jiao, Yuzhe Liu, Xi-Yong Yu, Xiangbin Pan, Yu Zhang, Junchu Tu, Yao-Hua Song, Yangxin Li

**Affiliations:** 1 Institute for Cardiovascular Science and Department of Cardiovascular Surgery, First Affiliated Hospital and Medical College of Soochow University, Collaborative Innovation Center of Hematology, Soochow University, Suzhou, Jiangsu 215123 P. R. China; 2 Department of Orthopedics, the Second Hospital of Jilin University, Changchun, Jilin 130000 P. R. China; 3 Key Laboratory of Molecular Target & Clinical Pharmacology and the NMPA State Key Laboratory of Respiratory Disease, Guangzhou Medical University, Guangzhou, Guangdong 511436 P. R. China; 4 Department of Structural Heart Disease, National Center for Cardiovascular Disease, China & Fuwai Hospital, Chinese Academy of Medical Sciences & Peking Union Medical College, Beijing, P. R. China; 5 Key Laboratory of Cardiovascular Appratus Innovation, Beijing, 100037 P. R. China; 6 Cyrus Tang Hematology Center, Collaborative Innovation Center of Hematology, Soochow University, National Clinical Research Center for Hematologic Diseases, the First Affiliated Hospital of Soochow University, Suzhou, P. R. China; 7 State Key Laboratory of Radiation Medicine and Protection, Soochow University, Suzhou, 215123 P. R. China

**Keywords:** Molecular medicine, Molecular biology

## Abstract

The ribosome is a multi-unit complex that translates mRNA into protein. Ribosome biogenesis is the process that generates ribosomes and plays an essential role in cell proliferation, differentiation, apoptosis, development, and transformation. The mTORC1, Myc, and noncoding RNA signaling pathways are the primary mediators that work jointly with RNA polymerases and ribosome proteins to control ribosome biogenesis and protein synthesis. Activation of mTORC1 is required for normal fetal growth and development and tissue regeneration after birth. Myc is implicated in cancer development by enhancing RNA Pol II activity, leading to uncontrolled cancer cell growth. The deregulation of noncoding RNAs such as microRNAs, long noncoding RNAs, and circular RNAs is involved in developing blood, neurodegenerative diseases, and atherosclerosis. We review the similarities and differences between eukaryotic and bacterial ribosomes and the molecular mechanism of ribosome-targeting antibiotics and bacterial resistance. We also review the most recent findings of ribosome dysfunction in COVID-19 and other conditions and discuss the consequences of ribosome frameshifting, ribosome-stalling, and ribosome-collision. We summarize the role of ribosome biogenesis in the development of various diseases. Furthermore, we review the current clinical trials, prospective vaccines for COVID-19, and therapies targeting ribosome biogenesis in cancer, cardiovascular disease, aging, and neurodegenerative disease.

## Introduction

The primary function of the ribosome is to synthesize proteins with mRNA as a template and amino acids as raw materials.^1^ Studies have demonstrated that the ribosome affects the rate of protein synthesis and plays a role in cell proliferation, differentiation, apoptosis, and transformation.^2–4^ When the ribosome is abnormal, it can severely affect the cell fate, causing various ribosome-related diseases such as COVID-19 virus infection, bacterial resistance, cardiovascular diseases (CVD), blood diseases, neurodegenerative diseases, and cancer.^5–7^ This review focuses on ribosome abnormalities to explore the molecular mechanisms of common diseases. Along this line, we discussed how the ribosome biogenesis pathway could be targeted for therapeutic purposes. For example, ribosomal protein S6 kinase 1 (S6K1) and nucleolin (Ncl) are drug targets for the treatment of cardiac hypertrophy and myocardial infarction (MI).

## Ribosomes and ribosome biogenesis

### Eukaryotic ribosome

Ribosomes comprise four ribosomal RNAs (rRNAs) and 80 ribosomal proteins (RPs). The eukaryotic ribosome includes two subunits, the 40S small subunit, and the 60S large subunit, which combine to form the 80S ribosome with translational activity.^8^ The 40S subunit is responsible for recognizing and binding messenger RNA (mRNA), while the function of the 60S subunit is to form peptide bonds. Hence, the ribosome is known as the “protein synthesis factory.”

**Fig. 1:**
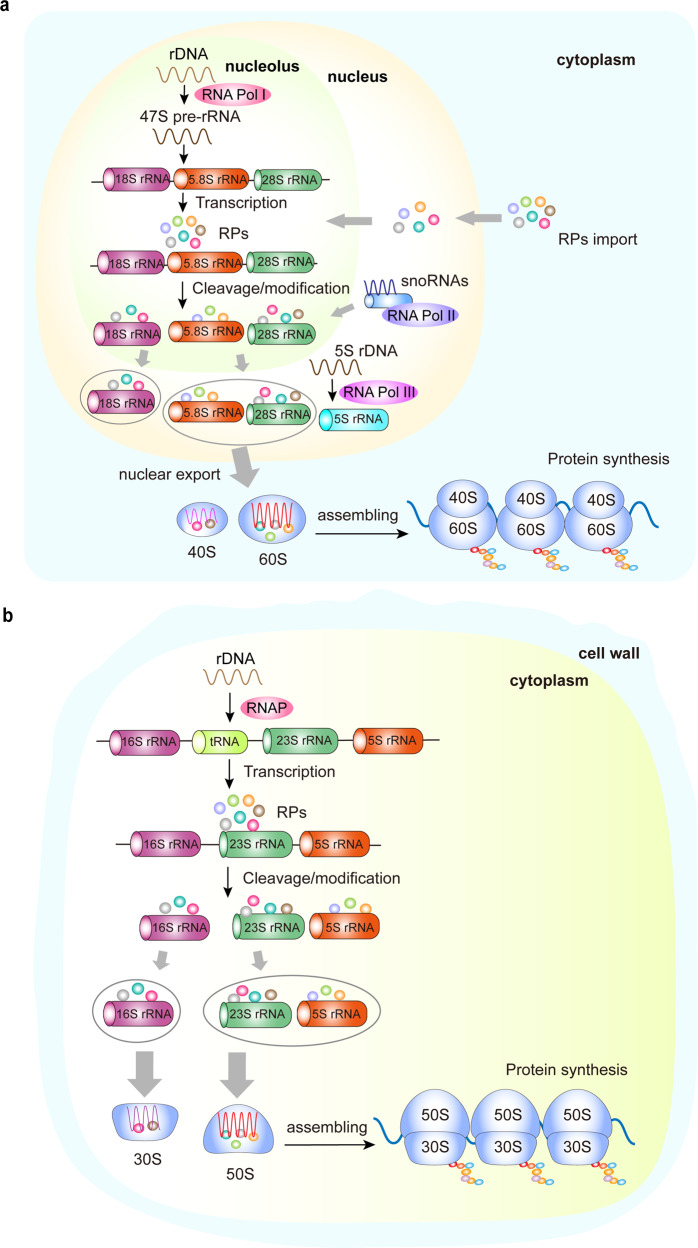
Schematic of ribosome biogenesis. **a** Eukaryotic ribosome biogenesis is a highly orchestrated process involving RNA Pol I, Pol II, and Pol III, which are responsible for transcribing rDNA to rRNA, producing 47S pre-rRNA in the nucleolus. The 4 rRNAs then assemble together with RPs to form a small ribosomal subunit (40S) and a large ribosomal subunit (60S). After assembly, the ribosome complex is exported from the nucleolus to the cytoplasm to form mature ribosomes for protein synthesis. **b** Bacterial ribosome biogenesis starts with the transcription of the precursors of 23S, 16S, and 5S rRNAs, and some tRNAs in the cytoplasm. The rRNAs then assemble with RPs to form a 30S small subunit and a 50S large subunit. After assembly, the ribosome complex forms mature ribosomes for protein synthesis. RP ribosome protein, RNA pol Ι/II/III RNA polymerase Ι/II/III, RNAP RNA polymerase, rDNA ribosomal DNA, rRNA, ribosomal RNA, tRNA transfer RNA

Ribosome biogenesis is the process of assembling the ribosome complex.^9^ This highly coordinated process is closely associated with protein synthesis, cell proliferation, differentiation, and apoptosis.^10^ Specifically, the process demands the organized synthesis of four rRNAs, 80 RPs, and ~70 small nucleolar RNAs.^11,12^ Within the nucleolus, the 47S pre-rRNA is transcribed from rDNA and cleaved to yield the 18S, 5.8S, and 28S rRNA. After synthesis in the cytoplasm, the RPs are imported to the nucleus to join the rRNAs. After modifications by snoRNAs, the rRNAs exit from the nucleolus. The 60S ribosome subunit is formed when the 5.8S, 28S rRNA is joined by the 5S rRNA in the nucleus and exported to the cytoplasm, while the 40S ribosome subunit is formed from the 18S rRNA. In the nucleolus of eukaryote cells, rRNA, RPs, small nucleolar RNAs, and other closely-related proteins participate in the generation of ribosome precursor 60S (pre-60S) and precursor 40S (pre-40S). The final ribosome assembly and maturation are completed when these molecules are exported to the cytoplasm (Fig. 1a).^13^

The maturation needs a series of chemical modifications of precursor rRNAs (pre-rRNAs). RNA polymerase I (Pol I) is required to process pre-47S transcripts into 5.8S, 18S, and 28S rRNA, and RNA polymerase III (Pol III) is responsible for the transcription of 5S rRNA.^13^ RPs are assembled into pre-rRNA transcripts and accelerate the formation of mature rRNA.^14,15^ Some RPs have other functions besides making ribosomes, including replication of DNA and cell division.^16,17^ Of note, ribosome dysfunction can lead to cellular dysfunction and eventually induce various diseases.

### Bacteria ribosome

Bacteria do not have a nucleus but have a cell wall, cell membrane, cytoplasm, and ribosomes. The bacterial ribosome is formed by the 30S, and 50S subunits, including three types of rRNA (23S, 16S, and 5S rRNA) and 54 RPs.^18^ Biogenesis of the bacterial ribosome starts when the primary rRNAs are transcribed, including 23S, 16S, and 5S rRNA precursors and some transfer RNAs (tRNAs).^19^ This process requires the participation of RNA polymerase (RNAP), a multi-subunit enzyme that synthesizes all types of RNA and catalyzes the transcription process of rDNA.^20^ Precursors of rRNAs and tRNAs fold into a unique structure, then excised from their primary transcripts. The 16S rRNA then assembles with RPs to form a 30S small subunit, while the 23S and 5S rRNAs assemble to form a 50S large subunit. The bacterial ribosomes are synthesized in the cytoplasm. After assembly, the ribosome complex forms the 70S mature ribosome rather than 80S due to tertiary structure (Fig. 1b).

Ribosome biogenesis is the process of creating ribosomes in a highly regulated manner. This process involves the synthesis of rRNAs and many RPs, which are required for proliferation and cell division.

## Signaling pathways involved in ribosome biogenesis

Ribosome biogenesis is regulated by several signaling pathways, among which mammalian targets of rapamycin (mTOR), myelocytomatosis oncogene (Myc), and noncoding RNA (ncRNA) are the most important regulators.

### mTOR

**Fig. 2:**
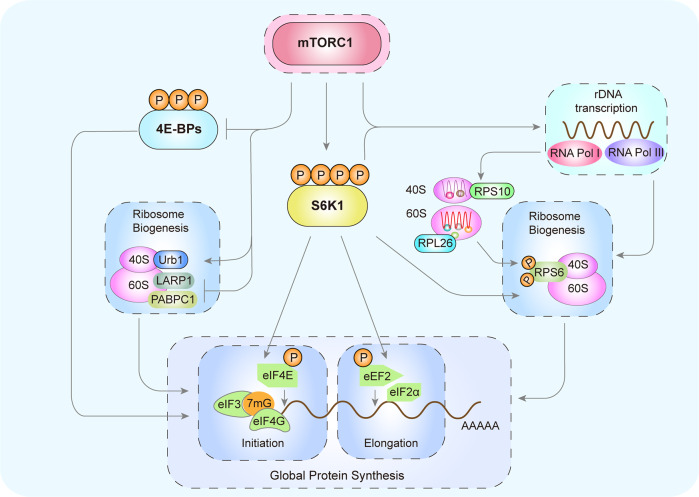
mTORC1 and ribosome biogenesis. Through phosphorylation, mTORC1 activates S6K1, which enhances rDNA transcription. mTORC1phosphorylates RPS6 to promote the synthesis of ribosomal 40S subunits, actively regulates the formation of RPS10 and RPL26, and promotes the initiation and elongation of mRNA through eIF4E and eIF2α, respectively. mTORC1 also phosphorylates and inactivates 4E-BPs to induce translation initiation. The RP assembly factor Urb1 is a downstream target of mTORC1 signaling. mTORC1 inhibits the binding of LARP1 and PABPC1 to promote mRNA translation and protein synthesis. mTORC1 mammalian target of rapamycin C1, rDNA ribosomal DNA, S6K1 S6 kinase 1, 4E-BP 4E-binding protein

mTOR is a serine/threonine kinase that regulates ribosome biogenesis. mTOR is assembled into multiple complexes, such as mTOR complex 1/2 (mTORC1/2).^21^ mTORC1 senses environmental cues to promote cellular anabolism and inhibit catabolism. The mTORC1 pathway regulates pre-rRNA transcription, rRNA synthesis, and RP expression. mTORC1 positively regulates rDNA transcription by activating S6K1 and promotes the synthesis of ribosomal 40S subunits through RPS6. mTORC1 promotes the initiation and elongation of mRNA through eIF4E and eIF2α. mTORC1 also induces translation initiation by inactivating 4E-BPs. The RP assembly factor Urb1 is a downstream target of mTORC1 signaling. mTORC1 promotes the translation of mRNA by inhibiting the binding of LARP1 and PABPC1 (Fig. 2).^22^

Ribosomal protein S6 kinase 1 (S6K1) is under the control of mTORC1 signaling.^23^ By activating the mTORC1/S6K1 pathway, checkpoint kinase 1 stimulates myocardial regeneration.^3^ Positive regulation of mTORC1 is required for normal fetal growth and development,^24^ and decreased expression of the RPs RPL26 and RPS10 in the placenta leads to intrauterine growth restriction in pregnant women. In addition, Urb1, an assembly factor of RPs, functions as a downstream target of mTORC1 signaling to promote the formation of the digestive systems in zebrafish.^25^ Therefore, the mTORC1/S6K1/RPL26/RPS10 ribosome biogenesis pathway plays an essential role in tissue development, growth, and regeneration.

mTORC1/S6K1 also regulates mRNA translation through multiple factors. The eukaryotic elongation factor 2 (eEF2) mediates ribosomal translocation.^26^ Its activity is inhibited by phosphorylation. S6K1 can enhance the activity of eEF2 by reducing its phosphorylation.^27^ The eukaryotic initiation factor 4G (eIF4G) phosphorylation stimulates protein synthesis. Vary et al.^28^ showed that mTORC1/S6K1 stimulates protein synthesis during skeletal muscle regeneration by increasing the phosphorylation of eIF4G. La-related protein 1 (LARP1) is an RNA-binding protein that regulates RP production. Smith et al.^29^ showed that inhibition of mTORC1 increases the binding of LARP1 to poly (A)-binding protein cytoplasmic 1 (PABPC1), resulting in translational inhibition of mRNA.

Moreover, 4E-binding protein 1 (4E-BP1), a key translation initiation factor downstream of mTORC1, is involved in metabolism, translation, and cell growth.^30^ Phosphorylation of 4E-BP1 results in the dissociation of 4E-BP1 from eIF4E, and association with eIF4G.^31^ eIF4G acts as a docking site to assemble other initiation factors, including eIF4A, which is an RNA helicase that interacts with eIF3 to recruit 40S ribosomes and other important initiation factors to the 5’ region of mRNA and begin protein synthesis.^32^

Importantly, inhibition of abnormal ribosome biogenesis using drugs that inhibit mTORC1/ribosomal protein S6 (RPS6) signaling is an effective strategy to treat cancer.^33^ Calvisi et al.^34^ showed that the activity of mTORC1 and RPS6 is higher in human hepatocellular carcinoma tissue than in the normal liver and promotes tumor progression. In addition, the mTORC1 inhibitor rapamycin significantly inhibits mTORC1 activity in cancer and RPS6 phosphorylation levels in bladder cancer. Similarly, rapamycin effectively inhibits the proliferation of lymphoma cells by inactivating the mTORC1/RPS6 signaling pathway and downregulating the expression of phosphorylated RPS6.^35^ PIK3CA mutations are common in some cancers. The mTORC1 inhibitor everolimus effectively inhibits cancer cell proliferation and differentiation in PIK3CA mutant colorectal cancer by reducing the phosphorylation level of RPS6.^36^

In summary, ribosome biogenesis regulated by mTORC1 plays a key role in tissue regeneration and cancer development.

### Myc

**Fig. 3:**
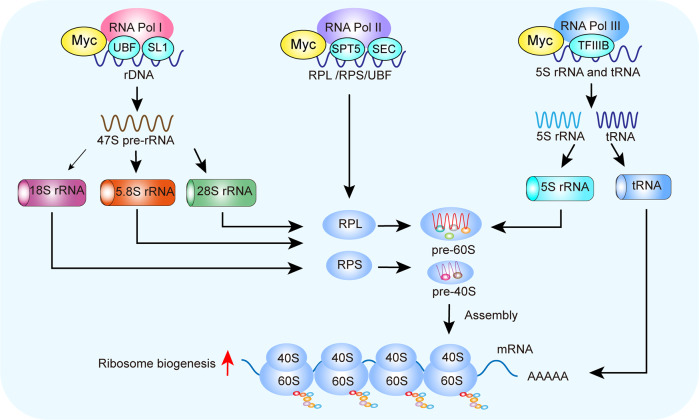
Myc regulates ribosome biogenesis. Myc directly regulates rRNA processing, ribosome assembly, translocation from the nucleus to the cytoplasm, and the early steps of mRNA translation. Myc upregulates the transcriptional levels of these factors by recruiting cofactors and remodeling chromatin structure. Myc promotes RNA Pol I-mediated rDNA transcription by binding to UBF and SL1. After transcription, the 47S pre-rRNA is processed into mature 5.8S, 18S, and 28S rRNA. The Myc–STP5/SEC complex stimulates rRNA processing and transcription of RPS and RPL for export in an RNA Pol II-dependent manner. Myc binds transcription factor IIIB (TFIIIB) and activates the transcription of 5S rRNA and tRNA mediated by RNA Pol III. Finally, Myc stimulates RP synthesis through these RNA Pol I–III pathways. Myc myelocytomatosis oncogene, SL1 selectivity factor 1, TFIIIB transcription factor IIIB, UBF upstream binding factor

Myc is one of the proto-oncogenes involved in abnormal ribosome biogenesis.^37^ An abnormal increase in nuclear size and quantity caused by Myc can be seen in most cancers. Mechanistically, Myc mainly regulates ribosome biogenesis through RNA Pol I–III-mediated rDNA transcription through UBF and SL1, rRNA processing and transcription of RPS and RPL, and the transcription of 5S rRNA and tRNA (Fig. 3).^38,39^

Myc promotes the transcription of rDNA in an RNA Pol I-dependent manner by recruiting upstream binding factor (UBF) and selectivity factor 1 (SL1) and enhancing their expression. SL1 is a transcription factor that facilitates the transcription of rRNA by recruiting RNA Pol I.^40^ Myc recruits RNA Pol I to target promoters by interacting with the TBP-associated factor, a component of the SL1 complex.^41^ CX-5461, a small molecule inhibitor of RNA Pol I, represses the proliferation of neuroblastoma cells^42^ and myeloma by inhibiting Myc expression and activating p53, leading to cancer cell apoptosis.^43^ Therefore, targeting ribosome biogenesis provides a new opportunity to treat tumors expressing high levels of Myc.

Upon binding to the initiation factor factor-IA (TIF-IA, also named RRN3), RNA Pol I is activated and recruited to the ribosomal DNA promoter.^44^ In medulloblastoma cells with high Myc expression, the increased rRNA synthesis is associated with increased expression of RNA Pol I-specific transcription initiation factor RRN3/TIF-IA.^45^ Interestingly, repression of rDNA transcription by CX-5461 is irreversible, and the RNA Pol I-RRN3 complex remains irreversibly locked in the pre-initiation complex even after drug removal. Furthermore, c-Myc overexpression is sufficient to stimulate the biogenesis 47S pre-rRNA, total RNA, and protein synthesis in skeletal muscle without activating mTORC1.^46^

Myc promotes uncontrolled cancer cell growth by enhancing RNA Pol II activity to maintain rapidly-progressive transcriptional elongation.^47^ The super-extension complex (SEC) is required for robust and efficient transcription by RNA Pol II. Abnormal SEC activity can induce cancer. In a Myc-driven mouse cancer model, disruption of the SEC protein downregulates Myc-dependent transcriptional programs in cancer cells, reducing ribosome biogenesis and slowing cancer progression.^48^ This suggests that small molecules targeting SEC can be used to treat Myc-induced cancer. Furthermore, overexpression of the human RNA Pol II-associated factor 1 complex (hPAF1C) is associated with the development of various cancers. Recent studies have demonstrated that hPAF1C is positively correlated with Myc, and non-small cell lung cancer patients with low hPAF1C expression have better overall survival. It has been shown that hPAF1C can promote lung cancer cell proliferation by enhancing Myc transcription.^49^

RNA Pol III employs specific transcription factors to drive the target genes.^50^ Myc increases the expression of 43% of the genes encoding RNA Pol III-specific subunits.^51^ Myc enhances the function of 5S rRNA and RNA pol III in various cell types.^52–54^ An increase in Myc expression results in enhanced 5S rRNA synthesis in cardiomyocytes and hepatocytes.^54,55^ The effect of Myc on RNA Pol III-dependent transcription is epigenetically regulated.^56^ Myc is also recruited to Pol III target genes through the RNA Pol III-specific transcription factor TFIIIB/C in different cell types.^57^ TFIIIC recruits Myc and the related histone acetyltransferase to promote binding to TFIIIB.^58^ Thus, Myc regulates ribosome biogenesis by interacting with RNA Pol III at the promoters of its target genes.

### Noncoding RNAs (ncRNAs)

**Fig. 4:**
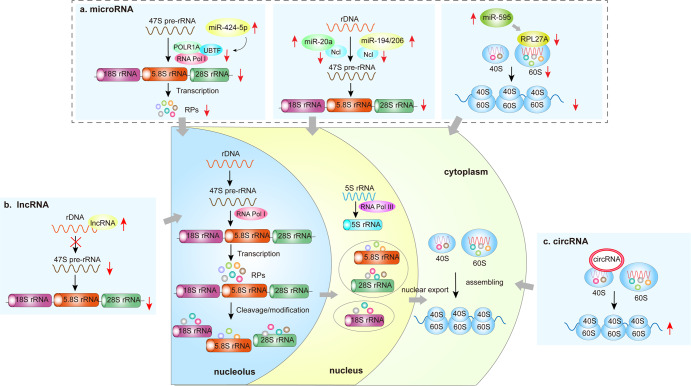
ncRNA and ribosome biogenesis. **a** There is a strong link between miRNA and ribosome biogenesis. miR-424-5p is elevated and targets the RNA Pol I pre-priming complex factors POLR1A and UBTF, which in turn downregulate mature rRNA levels. miR-20a, miR-194, and miR-206 inhibit Ncl expression by binding to the 3’UTR of Ncl, thereby affecting the subsequent splicing and processing of 47S pre-RNA. miR-595 inhibits ribosome biogenesis by reducing the expression of RPL27A and the synthesis of the mature 60S subunit. **b** Increased expression of lncRNA inhibits rDNA transcription through modification of rDNA chromatin, resulting in reduced pre-rRNA levels, mature rRNA levels, and overall translation. **c** circRNA recruits the 40S ribosomal subunit and initiates mRNA translation and protein synthesis. lncRNA long noncoding RNA, Ncl nucleolin, rDNA ribosomal RNA, pol Ι RNA polymerase Ι

Recent studies have shown that ncRNAs such as microRNAs (miRNAs), long noncoding RNAs (lncRNAs), and circular RNAs (circRNAs) are involved in regulating ribosome biogenesis (Fig. 4). miR-424-5p downregulates mature rRNA levels by targeting the RNA Pol I pre-priming complex factors POLR1A and UBTF. miR-20a, miR-194, and miR-206 affect processing of 47S pre-RNA by inhibiting Ncl expression. miR-595 inhibits the synthesis of the mature 60S subunit by reducing the expression of RPL27A. lncRNA inhibits translation by reducing pre-rRNA levels through modification of rDNA chromatin. circRNA recruits the 40S ribosomal subunit to facilitate the initiation of protein synthesis.

miRNAs are a type of short ncRNAs that regulate mRNA stability.^59^ Deregulation of miRNAs is involved in developing myelodysplastic syndrome (MDS) and Diamond-Blackfan anemia (DBA). The activation of miR-595, decreased ribosome protein RPL27A expression,^60,61^ and reduction of mature 60S subunits^60^ have been reported in patients with MDS, while the differential expression of some miRNAs has been found in the zebrafish model of DBA.^62^ Patients with chronic obstructive pulmonary disease show increased expression of miR-424-5p, which targets the RNA Pol I pre-priming complex factors POLR1A and UBTF. Upregulation of miR-424-5p is implicated in muscle wasting by inhibiting rRNA synthesis.^63^

lncRNAs also play important roles in ribosome biogenesis and serve as therapeutic targets or biomarkers in certain diseases. lncRNAs are >200 nt in length and highly diverse in characteristics, localization, and mode of action.^64^ Overexpression of lncRNA reduces pre-rRNA levels, mature rRNA, and overall translation.^65^ Some lncRNAs control the transcriptional activity of pre-rRNA by modifying rDNA chromatin, while small nucleolar RNA-terminal lncRNA is involved in nucleolar localization by enhancing pre-rRNA transcription.^66^ An lncRNA-mediated reduction of ribosome biogenesis may cause neurodegenerative diseases. Of note, the level of lncRNA is elevated in the mouse model of Alzheimer’s disease and is associated with nucleolar stress and reduced rRNA production.^67,68^

circRNAs are widely present in mammalian cells,^69^ and were previously thought to act only as miRNA sponges to refine the miRNA-mRNA axis.^70,71^ Emerging evidence suggests that circRNAs have protein-coding potential because they are associated with polysomes, including the initiation codon AUG and open reading frames.^72–74^ circRNA can engage the 40S ribosomal subunit and start protein translation.^75^ Holdt et al.^76^ found that circANRIL prevents atherosclerosis by inhibiting the proliferation of vascular smooth muscle cells via controlling rRNA maturation and ribosome biogenesis.

In summary, mTORC1 is involved in multiple steps in ribosome biogenesis, including the synthesis of rRNA, ribosome proteins, and the processing of precursors of rRNA. Myc overexpression increases ribosome biogenesis and is implicated in cancer cell growth. lncRNA-mediated reduction of ribosome biogenesis may cause neurodegenerative diseases. Deregulation of miRNAs contributes to the development of blood diseases. Thus, ribosome biogenesis is a potential druggable pathway for treating neurodegenerative and blood disorders.

## COVID-19 and ribosome biogenesis

**Fig. 5:**
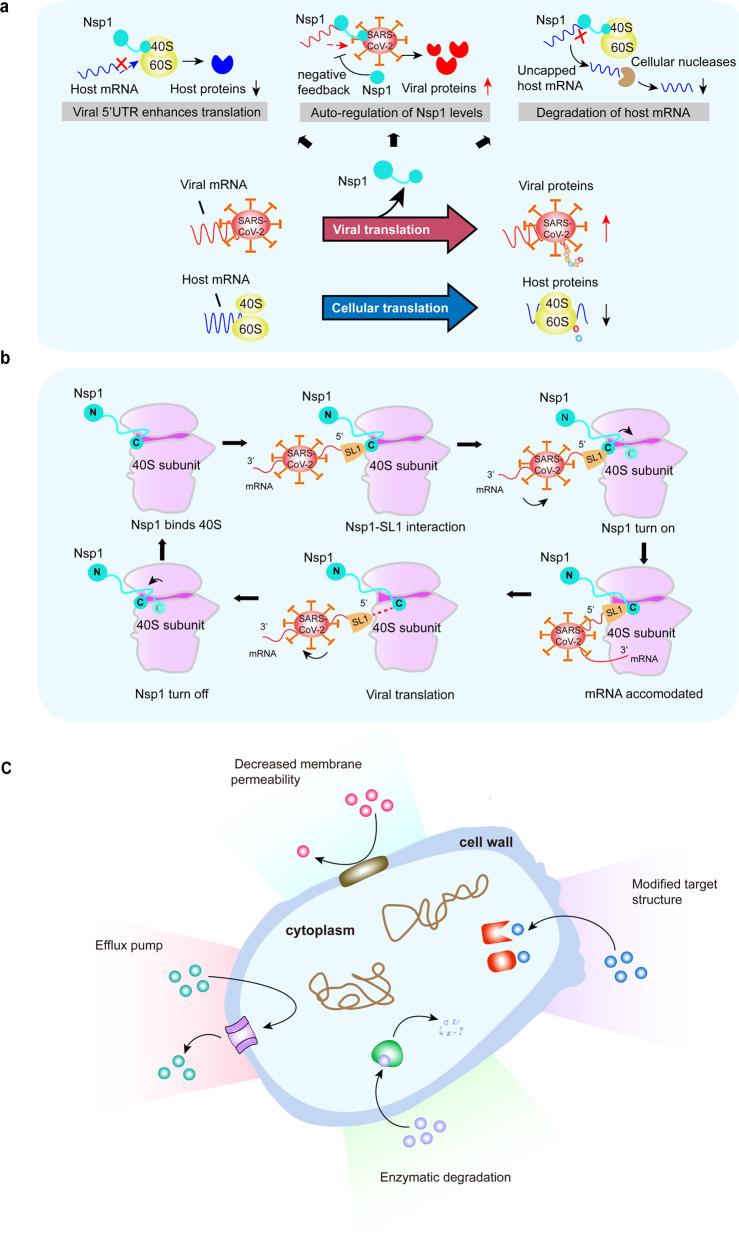
**a** SARS-CoV-2 regulates ribosome biogenesis in host cells through multiple pathways. Nsp1 is used by the SARS-CoV-2 virus to ensure its own replication and spread in the human host. The 5’UTR of viral Nsp1 is a key factor in directing ribosomes to viral transcripts and blocking host cell mRNA translation. Nsp1 blocks host mRNA translation and enhance the synthesis of viral proteins. Nsp1 alters the balance between viral and host cellular mRNAs through the cleavage of host mRNAs, leading to the degradation of host mRNAs by cellular nucleases. **b** Nsp1 acts as a gatekeeper to help SARS-CoV-2 evasion. Early in infection, Nsp1 binds to the 40S subunit with the carboxy-terminal domain of Nsp1. The viral mRNA transcript forms a translation initiation complex with the 40S-Nsp1 complex in its 5’UTR SL1. The carboxy-terminal domain of Nsp1 is removed to open the ribosome biogenesis channel for viral mRNA. The translation initiation, elongation, and termination further proceed. Upon termination, the viral mRNA is released, and the Nsp1 carboxy terminus refolds to prevent any de novo translation of cellular mRNA. **c** Mechanisms of bacterial resistance. Reducing the concentration of the harmful drug in the bacterium by decreasing the permeability of the bacterial outer membrane. The antimicrobial substance in the bacterial cell is removed by the efflux pumps. Mutation or modification of the target structure to reduce the affinity for antibiotics. Enzymatic degradation of antibiotics. Nsp1 nonstructural protein 1

The coronavirus disease 2019 (COVID-19) pandemic is caused by the severe acute respiratory syndrome coronavirus 2 (SARS-CoV-2) (Fig. 5a). SARS-CoV-2 regulates ribosome biogenesis in host cells through multiple pathways. Nsp1 is used by the SARS-CoV-2 virus to block the entry of host mRNA while inducing the cleavage of host mRNAs.

It was shown that SARS-CoV-2 infection affects ribosome biogenesis^77^ and leads to immune evasion and viral replication.^78^

### Immune evasion via cap structure of SARS-CoV-2 RNA

One of the mechanisms whereby the virus evades the immune system is through 2’-*O*-methylation of RNA by adding a methyl group to the 2’ hydroxyl of the ribose moiety, which is often found in the crucial regions of the ribosome.^79^ The methyltransferase activity of the RNA 2*’*-O-methyltransferase fibrillarin (FBL) is required for viral infection, which forms a strong rationale for developing FBL inhibitors as antiviral agents.^80^ More recent studies have demonstrated that SARS-CoV-2 evades the innate immune system by encoding its viral 2′-O-MTase, which is responsible for forming the cap structure at the 5′-end of SARS-CoV-2 RNA.^81,82^

Replication of SARS-CoV-2 RNA via IRES^83^: An internal ribosome entry site (IRES) is an element within RNA that initiates translation in a cap-independent manner. The location for IRES elements is often in the 5′UTR, but can occur elsewhere within the mRNA.^84^ Viral mRNAs use IRES to compete with host mRNA for ribosomes and translation factors. Small molecules that can change the structure of IRES may interfere with viral mRNA translation and block infection.^83^ The IRES elements in different RNA viruses differ in sequence, secondary RNA structure, and host factor requirement to recruit ribosome subunits.^85,86^ For example, type III IRES-mediated translation initiation requires the participation of multiple protein factors.^87,88^ These studies demonstrated that the IRES element mediates viral protein translation through interactions with various translation initiation factors in eukaryotic cells.

The SARS-CoV-2 genome is a positive-sense single-stranded RNA with a 5’ cap followed by an untranslated region.^89,90^ Due to the 5’ end cap and 3’ poly(A) tail, SARS-CoV-2 directly utilizes a cap-dependent mechanism to initiate translation after infecting host cells.^91^ In addition, the 5’UTR contains a region with high CG content, which can form an IRES to recruit host ribosomes to translate its RNA in a cap-independent manner.^92^ By taking advantage of these features, SARS-CoV-2 can replicate efficiently in host cells.^93^ With the assistance of IRES, SARS-CoV-2 can efficiently compete with the host cells for initiation factors and translation machinery to produce viral proteins. Therefore, drugs targeting IRES may block viral infection. Prunin is one such compound that inhibits the IRES activity of human enterovirus A71, and has shown promising results in treating hand, foot, and mouth disease.^94^ Islam et al.^95^ detected three specific mutations in the IRES that can destroy its secondary structure, thereby reducing the function of the virus. These results may guide the development of drugs targeting the IRES to slow the spread of SARS-CoV-2.

In summary, both cap-dependent and cap-independent mechanisms are utilized by the SARS-CoV-2 genome to translate viral proteins. Further study of the activity, mechanism of action, and inhibition mode of IRES elements in the 5’UTR of the SARS-CoV-2 genome can provide valuable information for developing therapeutic drugs.

### Production of viral proteins via ribosome-frameshift

Viruses must use the host cell’s ribosomes to synthesize their proteins. Normally, the ribosomes move along the mRNA, reading three bases at a time and synthesizing an in-frame protein. Sometimes, ribosomes miss a base or two, generating a frameshift, resulting in a dysfunctional protein. The coronaviruses rely on frameshift to hijack the cell’s translation process and produce viral proteins.^96^ Using cryo-electron microscopy, Bhatt et al. discovered that the viral RNA promotes a frameshift by forming a pseudoknot structure at the entrance of the ribosomal mRNA channel. It has been shown that merafloxacin reduces the titer of new coronavirus by inhibiting the frameshift efficiency.^97^

### Nsp1 is a potential vaccine target

The nonstructural proteins (Nsp1–Nsp16) facilitate virus replication by regulating the RNA-directed RNA polymerase and helicase.^98^ The Nsp1 of SARS-CoV-2 has been identified as a virulence factor because it helps to produce viral protein while inhibiting the protein synthesis of infected cells. Using high-resolution cryo-electron microscopy, Thoms et al.^99^ demonstrated that the Nsp1 protein could shut down the translation of host mRNA by preventing the binding of mRNA with the ribosomal 40S subunit. Understanding the Nsp1 inhibitory mechanism has been helpful for the design of anti-SARS-CoV-2 drugs. In addition, Nsp1 inhibits intracellular antiviral defense signaling pathways by blocking the retinoic acid-inducible gene I-dependent innate immune responses.^99–101^

Moreover, Nsp1 acts as a gatekeeper to restrict translation to only viral transcripts. The release of the Nsp1 is controlled by viral SL1. In coronaviruses, the viral transcripts contain three hairpins SL1, SL2, and SL3, at the 5′end.^102^ SARS-CoV-2 also contains these hairpins.^91^ More specifically, SL1 acts on the Nsp1 carboxy-terminal domain to allow viral RNAs to enter the ribosome while Nsp1 is still bound on the ribosome. SL1 plays a critical role in the completion of the viral infection. SL1 is regarded as the real "Soft Rib" of SARS-CoV-2.^103^ Therefore, targeting SL1 during SARS-CoV-2 infection would be a precise approach to shut down viral translation (Fig. 5b). SL1 acts as a switch to shift the translation machinery to the virus through its interaction with Nsp1.^104^

Other Nsps as potential vaccine targets: Upon infection, the Nsps produced by SARS-CoV-2 inhibit interferon response by blocking the ribosome channel to disrupt protein translation (Nsp1), suppressing global mRNA splicing (Nsp16), and blocking protein trafficking to the membrane (Nsp8 and Nsp9).^105^ Moreover, Nsp6 and Nsp13 antagonize IFN-I production by suppressing interferon regulatory factor 3.^106^ Thus, IFN can be used as an indicator of disease severity and potential treatment to enhance the host immune response to viral infection.^107^

Through RNA sequencing, ribosome profiling, and metabolic labeling of newly synthesized RNA, Finkel et al.^108^ showed that although viral transcripts are not preferentially translated, the virus dominates the mRNA pool in infected cells by inducing the degradation of host mRNAs. Virus inhibits the translation of innate immune genes by blocking nuclear mRNA export, preventing cellular mRNA from reaching the ribosomes.

Another study showed that herpes simplex virus type 1 (HSV-1) utilizes γ34.5 to promote its replication by inducing the cytoplasmic translocation of nucleolar protein NOP53, which in turn facilitates the recruitment of PP1αto dephosphorylate eukaryotic initiation factor eIF2α for viral translation.^109^

In contrast to the common belief that viral replication relies on increased ribosome abundance, Bianco et al.^77^ showed that human cytomegalovirus (HCMV) productive growth was enhanced by restricting ribosome biogenesis. The impairment of ribosome biogenesis leads to reduced interferon beta (IFNB1) mRNA accumulation, leading to enhanced replication of HCMV.

The clinical application of ribosome protein (RP): To investigate whether ribosome biogenesis affects the innate immune response in patients with SARS-CoV-2 infection, Yang et al.^110^ compared the cellular and molecular characteristics of patients with long-duration of viral shedding (LD) with those of short-duration patients. They demonstrated that LD is associated with reduced levels of RP genes, decreased numbers of natural killer cells, and CD14^+^ monocytes but increased regulatory T cells. These results suggest that immunosuppression and low expression of RPs are associated with the persistence of SARS-CoV-2 infection in COVID-19 patients. However, it needs to be clarified whether the decreased level of RPs in SARS-CoV-2/Nsp1/RP is a cause or a consequence of viral persistence and whether specific RPs can be used as a clinical indicator for virus persistence.

In summary, these studies uncovered how viruses promote their growth by taking over the translation machinery while suppressing the host’s innate immune response.

## Bacteria and ribosome biogenesis

The number of antibiotic drugs approved by the Food and Drug Administration (FDA) of the United States has been increasing in recent years.^111^ However, after repeated use, bacteria become resistant to treatment. The rate of the development of drug-resistant bacteria is much faster than antibiotic development.^112,113^ With the in-depth study of ribosome structure, antibiotics targeting bacterial ribosomal protein synthesis have been developed.

### Mechanism of ribosome-targeting antibiotics

#### Structural basis of the bacterial ribosome

The bacterial ribosome comprises a 30S small subunit and a 50S large subunit, including 3 rRNAs and 54 ribosomal proteins.^18,114^ Antibiotics targeting the 50S subunit act on the peptidyl transferase center (PTC), the GTP hydrolase association center, the nascent peptide chain channel, or interfere with protein synthesis by incorporation into the extended peptide chain.^115^ For the 30S subunit, antibiotics prevent protein synthesis by inhibiting the formation of the ribosomal initiation complex, interfering with tRNA binding, and interfering with tRNA site transfer during translation.^19,116^

#### Antibiotics targeting tRNA

Tetracycline is a broad-spectrum antibiotic that blocks bacterial protein synthesis.^117^ It binds to the 30S ribosome subunit and prevents aminoacyl-tRNA binding to the ribosome A-site.^112,118,119^ Although tetracycline is widely used in the clinic, drug resistance due to mutations within the ribosomal binding sites limits its clinical efficacy.^120^ Due to the emergence of drug-resistant bacteria, the second (doxycycline) and third generation (glycylcyclines) of tetracyclines were produced. The glycylcycline antibiotic tigecycline has a similar mechanism of action as tetracycline but with higher affinity to the 30S ribosome, thus, blocking bacteria protein synthesis more efficiently.^121–123^

Aminoglycoside antibiotics target the aminoacyl group of the 16S ribosomal RNA on the 30S ribosome subunit, preventing the correct positioning of aa-tRNA at the A and P positions, resulting in a misreading of the genetic code and abnormal protein synthesis.^119^ Macrolide antibiotics target bacterial translation by binding to the P site in the 50S ribosome subunit. Mupirocin blocks bacterial protein synthesis by inhibiting isoleucine tRNA synthetase.^124^ Under the action of these antibiotics, the bacterial translation process is impaired, resulting in the death of the bacteria.^125,126^

#### Targeting peptidyl transferase center

The peptidyl transferase is an aminoacyl transferase that catalyzes the reaction to form peptide bonds between two adjacent amino acids. This is a process of adding an amino acid to the growing polypeptide chain during protein synthesis. The peptidyl transferase center (PTC) is situated in the large subunit of the ribosome. Chloramphenicol and macrolides are two examples of antibiotics targeting the PTC. Chloramphenicol prevents peptide bond formation by binding to the 23S rRNA of the 50S ribosome subunit, whereas macrolides do so by binding to the P site of the 50S subunit. The position where chloramphenicol binds to PTC overlaps with a part of the acyl group at the A-site of tRNA (Cam1).^127,128^ The binding site (Cam2) of chloramphenicol to the 50S subunit of the halophilic archaea is located deeper in the exit channel, overlapping the erythromycin binding site.^129^ The binding site of chloramphenicol to the archaeal 50S subunit is not in Cam1, which is in line with the finding that higher concentrations of chloramphenicol are needed to inhibit archaeal growth than bacterial growth. Chloramphenicol and erythromycin directly inhibit the biosynthesis of the 50S subunit.^130^ Erythromycin does not inhibit PTC but interferes with aminoacyl translocation, preventing the move of tRNA from the A-site to the P site of the ribosome.

### Molecular mechanisms of bacterial resistance

At present, it is generally believed that there are four main categories of molecular mechanisms by which bacteria develop drug resistance:^131,132^ (1) Reduction of intracellular drug concentration; (2) Modification of drug target due to mutation or modification of target genes; (3) Overexpression and protection of target genes; (4) Inactivation of the drug due to hydrolyzation (Fig. 5c). Thus, drug resistance is achieved by reducing the permeability of the bacterial outer membrane, decreasing the concentration of the antimicrobial substance by efflux pump, reducing the affinity for antibiotics through mutations, and degrading the antibiotics.

#### Reducing intracellular drug concentration

Gram-negative bacteria contain an outer membrane that protects the bacteria from external toxic agents. Therefore, Gram-positive bacteria are more susceptible to antibiotics than Gram-negative bacteria.^133^ Another way of keeping drug concentration low is to increase its efflux by acquiring genes encoding an efflux pump through chromosome mutation. The upregulation of the efflux pump causes drug resistance to most ribosome-targeting antibiotics.

#### Mutation or modification of target gene

Mutations in target genes can lead to reduced drug affinity.^115,116^ The ribosome-targeted antibiotics interact with rRNA, so mutations in rRNA nucleotides can change the conformation of the drug-binding site, resulting in tolerance. Most bacteria have more than one copies of rRNA operons, which means that only the same mutations that occur in all of the operons will the bacteria become resistant to the drug, which is a rare situation. It mainly occurs in *Mycoplasma pneumoniae* and *Mycobacterium tuberculosis* because these pathogens only have one or two rRNA operons. rRNA methyltransferases—mediated methylation of rRNA is one of the most common modifications. The methyltransferase KgmA and KamA–mediated methylation confer aminoglycoside tolerance.^134^ Cfr methylates nucleotide A2503 of 23S rRNA,^135^ a modification that promotes bacterial resistance to chloramphenicol, SA antibiotics, lincosamides,^136^ and some intracyclic macrocyclic Ester antibiotic resistance.^137^ Methylation of A2058 of 23S rRNA is responsible for bacterial resistance to macrolides, lincosamide, and streptogramins B.

#### Overexpression and protection of target genes

Overexpression of target genes leads to resistance to certain antibiotics because these target genes can sequester drugs. Antibiotic resistance caused by target gene overexpression has not been demonstrated in vivo, but they have been confirmed by in vitro experiments. Overexpression of 16S rRNA h34 resulted in bacterial resistance to spectinomycin.^138^ Likewise, overexpression of EF-Tu restored protein translation in the presence of EF-Tu inhibitors.^139^

#### Hydrolyzing or modifying enzymes that inactivate antibiotics

Certain enzymes encoded by bacteria are capable of modifying or degrading antibiotics.^140,141^ These enzymes include hydroxylases (beta-lactamases, esterases, epoxide hydroxylase) (phenicols), transferases (acetyltransferases, phosphotransferases, nucleotidyltransferases, glycosyltransferases, ADP-ribosyltransferases, S-transferases) (aminoglycosides and macrolides),^142,143^ and redox enzymes (monoxygenases, lyases) (tetracycline^144^and tigecycline^145^). Esterases EreA and EreB hydrolyze macrolide ester bonds, leading to resistance to macrolide antibiotics.^141^ For aminoglycosides, drug inactivation due to modification of their chemical structure by bacterial enzymes is the main mechanism of drug resistance. The amide and hydroxyl groups of aminoglycosides can be modified by acetyltransferase, nucleotidyl transferase, and phosphotransferase.

Ribosome biogenesis is an attractive target for developing new antibiotics. Antibiotic resistance mechanisms include degradation and efflux of the drug and mutation of target genes. Future work should focus on developing antibiotics targeting new sites on the ribosome.

## Ribosomes and cardiovascular diseases (CVDs)

In 2020, the World Health Organization proclaimed that the mortality caused by CVDs is significantly higher than that caused by tumors and other diseases.^146^ Many studies have pointed out that ribosome dysfunction triggers various CVDs.^3,4^ Here, we discuss the specific regulatory mechanisms of rDNA, rRNA, RPs, and Ncl in CVDs (Table 1).

**Table 1:** Ribosome-related factors and CVD

Factors	Types	Targets	Function	Ref.
rDNA	Cardiomyocyte	p-UBF↑	Promotes cardiac hypertrophy	^201–204^
	Cardiomyocyte	Ncl↑	Inhibits myocardial infarction	^235–237^
	Cardiomyocyte	Ncl↑	Promotes cardiomyocyte differentiation	^166,169,170^
rRNA	Cardiomyocyte	S6K1↑	Promotes cardiac hypertrophy	^161^
	Cardiomyocyte	RNA Pol I↑	Promotes cardiac hypertrophy	^160^
	Cardiomyocyte	rDNA↑	Promotes cardiac hypertrophy	^151,161^
RP	Cardiomyocyte	RPL9/26↓	Promotes myocardial infarction	^168^
	Macrophages	RPL13↑	Inhibits atherosclerosis	^181–183^
	Blood cells	RPL9/23/35, RPS7↓	Promotes atherosclerosis	^184^

### Ribosome and cardiac hypertrophy

Cardiac hypertrophy is an adaptive response that may occur after pressure or volume overload, inflammatory cardiomyopathy, and long-term stimulation, ultimately leading to heart failure.^147,148^ However, the molecular mechanisms underlying cardiac hypertrophy are currently unclear. Recent studies have suggested that ribosome biogenesis plays an important role in cardiac hypertrophy by promoting or inhibiting the pathological process.^149,150^

The rDNA transcription rate is positively correlated with the degree of phosphorylation of RNA Pol I and the upstream binding factor (UBF). Studies have shown that the hypophosphorylated form of UBF blocks rDNA transcription by disrupting the UBF/SL1 complex.^151^ Others reported that the proliferation rate of cultured neonatal cardiomyocytes is correlated with the phosphorylation of UBF.^152^ Endothelin-1-induced cardiac hypertrophy is associated with UBF hyperphosphorylation.^153^ Moreover, enhanced rDNA transcript levels are associated with increased UBF protein levels under α1 adrenergic receptor- or stress-load-induced cardiac hypertrophy. Further studies have confirmed that high UBF expression and activity stimulate ribosome biogenesis during cardiac hypertrophy.^154,155^

rRNA not only acts as the central scaffold for ribosomal subunits but also serves as the center for catalytic activity in ribosome biogenesis.^156,157^ The abnormal pre-rRNA processing causes defects in ribosome structure and function, disrupts cardiac protein balance, and induces cardiac hypertrophy. Rackham et al.^158^ reported that when the endoribonuclease component of the RNase P complex, MRPP3, is knocked out in the heart, the mice develop severe cardiomyopathy and the lifespan is shortened. The loss of MRPP3 causes defects in rRNA processing, leading to the production of immature rRNAs and a reduction of RPs. Their findings revealed that rRNA processing acts as a "bridge" to link transcription and translation by regulating ribosome assembly. In addition, RNA Pol I is primarily responsible for the processing of pre-47S transcripts into 5.8S, 18S, and 28S rRNA, and then generating mature rRNAs.^15^ Studies have demonstrated that TAF1A gene mutation of RNA Pol I cause gene-specific nucleolar defects and impair rRNA synthesis in cardiomyocytes, eventually leading to decreased ventricular systolic function, dilated cardiomyopathy (DCM), and heart failure.^9,159,160^

**Fig. 6:**
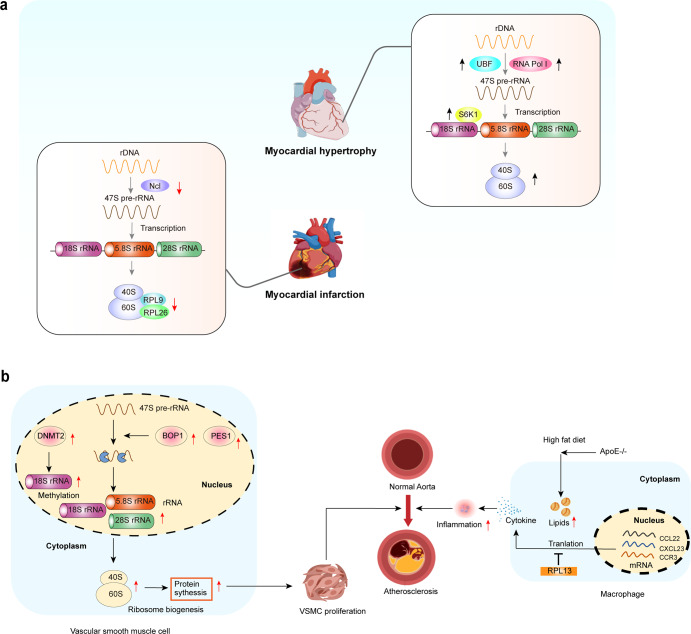
Ribosomes and CVD. **a** (i) Ribosome dysfunction and cardiac hypertrophy. Increased UBF activity and abnormal activation of RNA Pol I and S6K1 induce cardiac hypertrophy. (ii) Ribosome dysfunction and MI. Downregulation of Ncl, rRNA, RPL9, and RPL26 leads to the failure of pre-rRNA processing, ultimately causing ribosome dysfunction and MI. **b** Ribosomes and atherosclerosis. Left: The over-activation of ribosome biogenesis results in abnormal proliferation of VSMCs and atherosclerosis. The expression of BOP1 and PES1 is increased in atherosclerotic patients, and both promote rRNA maturation by promoting pre-rRNA splicing and ribosome biogenesis. Right: RPL13 inhibits macrophage-induced inflammation and atherosclerosis. A high-fat diet promotes atherosclerosis in ApoE^−/−^ mice, accompanied by higher levels of inflammatory cytokines. RPL13 inhibits the inflammatory response by inhibiting the translation of inflammatory genes such as CCL22, CXCL13a, and CCR3. BOP1 blocking of proliferation 1, PES1 pescadillo homolog 1

S6K1-dependent activation of RP synthesis is crucial for cardiomyocyte proliferation and growth. S6K1 promotes rDNA transcription in cardiomyocytes, accelerating the pathological evolution of cardiac hypertrophy.^161^ This suggests that S6K1 is a key factor in regulating ribosome biogenesis and cardiomyocyte growth. The above studies suggest that aberrant activation of S6K1 increases protein synthesis and growth in hypertrophic cardiomyocytes. S6K1 is an important drug target for the treatment of cardiac hypertrophy (Fig. 6a). Thus, abnormal upregulation of UBF, RNA Pol I, and S6K1 induces cardiac hypertrophy.

### Ribosomes and myocardial infarction (MI)

MI is caused by occlusion of the coronary artery. Although intracoronary vascular surgery can prolong the life of patients,^162^ postoperative ischemia-reperfusion injuries can cause further damage to the heart. Moreover, insufficient cardiac regeneration eventually leads to ventricular remodeling and heart failure.^163,164^ Many studies have confirmed that ribosome dysfunction plays an important role in MI.^165–167^ Downregulation of Ncl, rRNA, RPL9, and RPL26 is involved in MI-induced ribosome dysfunction (Fig. 6a).

Recent bioinformatics analysis showed that the expression levels of ribosomal proteins L9 (RPL9) and L26 (RPL26) are downregulated in patients with MI compared with controls.^168^ Therefore, RPL9 and RPL26 are potential targets for diagnosing or treating MI.

Ncl has many functions in cell survival and growth pathways. Some studies have found that knockout of Ncl in mice aggravates cardiac function after MI and reduces the survival rate. Mechanistically, Ncl improves cardiac function during MI by enhancing M2 macrophage polarization.^169^ In mouse models of MI, autophagy-related gene deletion or drug inhibition aggravates cardiac dysfunction and myocardial remodeling.^170^ Deng et al.^166^ found that the Ncl/autophagy signaling pathway is upregulated in the infarcted myocardium. Moreover, Nicorandil treatment affects the TGF-β/Smad signaling pathway by up-regulating the Ncl/autophagy axis, reducing cardiac remodeling after MI, and improving cardiac function. These studies suggest that Ncl is a potential target for the treatment of MI.

### Ribosomes and atherosclerosis

Atherosclerosis is caused by lipid deposition in the intima of the arterial wall and increasing its thickening. This is the common pathological basis of CVDs, such as MI and coronary heart disease.^171^

Abnormal proliferation of vascular smooth muscle cells (VSMCs) accelerates the process of atherosclerosis by promoting arterial intimal hyperplasia. Therefore, inhibiting the abnormal proliferation of VSMCs is the current method to inhibit neointimal hyperplasia.^172–174^ Ribosome biogenesis regulates cell proliferation and differentiation. It is known that ribosome biogenesis includes three key steps: rRNA synthesis, ribosomal protein (RP) synthesis, and their assembly into the mature ribosome.^175,176^ The maturation of rRNA during ribosome assembly is closely associated with the PES-BOP1-WDR12 (PeBoW) complex, which binds to pre-rRNA and promotes 47S pre-rRNA into mature 28S and 5.8S rRNA. The core regulator of the PeBoW complex may be involved in the production of 28S and 5.8S rRNA.^177^ Jia et al.^177,178^ found that BOP1 is increased in the coronary arteries of patients with atherosclerosis and is accompanied by the neointimal hyperplasia caused by VSMC proliferation (Fig. 6b). Therefore, increased expression of BOP1 and PES1 promotes ribosome biogenesis leading to abnormal proliferation of VSMCs and atherosclerosis. Holdt et al.^76^ confirmed that circARNL occupies the pre-rRNA site of another member of the PeBoW complex, PES1, and inhibits the cleavage of pre-rRNA by exonuclease, thereby inhibiting the proliferation of VSMCs by inhibiting ribosome biogenesis and delaying the atherosclerotic process. Importantly, it has been reported that curcumin effectively inhibits the proliferation of VSMCs and slows the development of atherosclerosis. The mechanism is that curcumin promotes DNMN2 expression, which in turn elevates the levels of 18S rRNA methylation.^179^ The above reports suggest that suppressing ribosome biogenesis may be a new approach for inhibiting the proliferation of VSMCs.

Atherosclerosis is also a chronic inflammatory disease, as an inflammatory response accompanies its onset.^180^ Macrophages are one of the cells responsible for atherosclerosis; they form foam cells by phagocytosing lipid droplets and accelerating the progression of atherosclerosis.^181,182^ Basu et al.^183^ reported that macrophage-specific deletion of RPL13 increases the susceptibility of ApoE knockout mice to high-fat diet-induced atherosclerosis. The protective effect of RPL13 on atherosclerosis may be related to its translational silencing activity: the deletion of RPL13 promotes the translation of mRNAs for CCL22, CXCL13, and CCR3 in macrophages, causing increased expression of inflammatory cytokines. In summary, RPL13 inhibits macrophage-induced inflammation and atherosclerosis by inhibiting the translation of inflammatory genes such as CCL22, CXCL13a, and CCR3.

Since atherosclerosis is a chronic process, the time from its onset to its detection may take a long time. The use of ribosome biogenesis as a predictive factor for atherosclerosis has emerged. Wang et al.^184^ analyzed the plasma samples of patients with familial hypercholesterolemia and found that RPL9, RPL35, RPS7, and RPL23 showed a downward trend. The expressed genes were mainly involved in the ribosomal and oxidative phosphorylation pathways. Jiménez et al.^185^ found that m6A in the 18S rRNA component is significantly reduced in both early and late atherosclerosis samples by mass spectrometry, suggesting that 18S rRNA has abnormal methylation modification in early and late atherosclerosis. Therefore, various components of the ribosome may be used for the early detection of atherosclerosis.

### Nucleolin (Ncl) and CVD

Ribosome biogenesis begins in the nucleolus, where different rRNA subunits are transcribed as a polycistronic transcript by RNA polymerase I. More than 300 non-ribosomal factors, including nucleolin, regulates pre-rRNA transcription and ribosome assembly.^186^ Due to the complex nature of the ribosome biogenesis process, misassembly of the ribosomes may occur. To prevent the build-up of incorrectly folded ribosomes, a quality control mechanism is in place to detect and degrade the non-functional ribosomes, and this process is regulated by non-ribosomal proteins.^187^ Nucleolin is also present on the cell surface and is overexpressed in cancer cells.^188^

Ncl regulates cellular functions by participating in multiple stages of ribosome biogenesis, such as rDNA transcription, increased RNA Pol I transcriptional activity, and ribosome assembly.^189,190^ In addition, as a shuttle protein, the correct subcellular localization of Ncl is critical for its biological function, and the abnormal localization of Ncl is involved in various pathological processes.^190^ Recent studies suggest that abnormal Ncl expression is involved in the development of CVD, including MI^191^, heart failure (HF)^192^, and atherosclerosis.^193^

The protein level of Ncl is significantly decreased during MI, while the overexpression of Ncl reduces the infarct area and cell death in rat hearts.^194^ Cardiomyocyte-specific Ncl transgenic mice are resistant to doxorubicin-induced cardiotoxicity, indicating the cardioprotective effect of Ncl in this process.^195^ Of note, the heart of Ncl-deficient fish exhibits a severe cardiomyocyte defect, cardiac development disorder, and abnormal ventricular remodeling and dysfunction; the mechanism involves the reduction of rRNA transcription and heterochromatin caused by reduced Ncl.^196^ Knockdown of Ncl expression in neonatal rat ventricular myocytes leads to an increased expression of heterochromatin marker H3K9Me3 and a decrease in the transcript levels of pre-rRNA and 18S rRNA.^197^

Current studies confirm that Ncl can protect against MI through myocardial ischemic preconditioning (IP).^191^ IP is a short period of myocardial ischemia/reperfusion (I/R), significantly reducing the damage caused by subsequent long-term I/R, and is also a powerful endogenous heart protection mechanism.^198,199^ Jiang et al.^200^ found that Ncl is an important endogenous cardioprotective factor in myocardial IP. Ncl binds to the 3’UTR of HSPA1A mRNA, a member of the heat-shock protein (HSP) family, to upregulate the expression of HSPA1A by stabilizing its mRNA. As a phosphorylated protein, Ncl participates in rRNA synthesis and cell proliferation.^190^ Tong et al.^167^ demonstrated that threonine phosphorylation at positions 76 and 84 is essential for Ncl to reduce caspase-3 activity and inhibit cardiomyocyte apoptosis. This study also found that Ncl undergoes phosphorylation modification after translocation from the nucleus to the cytoplasm.

Macrophage phenotype-switching between the pro-inflammatory M1 and the anti-inflammatory M2 phenotype exhibit plasticity during the post-MI inflammatory response and tissue repair.^201^ Using a mouse MI model, Tang et al.^169^ found that the expression of Ncl mRNA and protein decreased gradually from day 3 after MI, then increased gradually, reaching a peak on day 7. The macrophages in the myocardium switch from the M1 phenotype 2 days after MI to the M2 phenotype 5 days after MI, suggesting a potential correlation between Ncl expression and macrophage phenotype-switching. Mechanistically, Ncl promotes the polarization of M2 macrophages by binding to the 5’ UTR or translation region of Notch3 and STAT6 mRNA to promote their stabilization.

The main feature of atherosclerosis is the formation of fat-rich plaques in medium and large vessels.^202^ The progression of the disease is linked to the recruitment of monocytes that differentiate into macrophages, which subsequently absorb lipids to become foam cells that produce inflammatory cytokines. Thus, rapid clearance of foam cells can inhibit the inflammatory response and ultimately delay plaque development.^203,204^ Ncl plays an important role in regulating the progression of atherosclerosis.^203^ Li et al.^205^ reported that macrophage transformation into foam cells is associated with decreased expression of Ncl protein. Ncl enhances the stability of ABCA1, which promotes cholesterol efflux and inhibits lipid accumulation and the formation of foam cells. It has also been shown that under normal conditions, the interaction between Ncl with Dnm3os prevents its enrichment to histone H3K9ac on the promoters of pro-inflammatory genes such as IL6.^193^ However, Sun et al.^206^ showed that oxidized low-density lipoprotein (oxLDL), one of the inducers of atherosclerosis, upregulates Ncl mRNA and protein expression in vascular smooth muscle cells (VSMCs) in a dose-dependent manner. The abnormal proliferation of VSMCs is the pathological basis for the development of atherosclerosis. Ncl promotes VSMC proliferation and cell cycle changes under oxLDL treatment. Under normal circumstances, Aurora B is mainly located in the nucleus and is dynamically expressed in the G2-M phase. Aurora B is associated with Ncl proteins, and it is speculated that the combination of the two promotes cell proliferation.

In summary, ribosome biogenesis plays an essential role in developing CVDs. The specific functions of ribosome biogenesis regulators, such as Ncl, in different cardiovascular cells and diseases deserve further study.

## Ribosomes, aging, and neurodegenerative diseases

Aging is a complex multifactorial, and irreversible biological process characterized by the degeneration of cellular, tissue, and organ functions over time. Among the various molecular mechanisms of aging, deterioration of the rate of protein synthesis and the function of ribosomes plays a central role.^207–209^

### The evidence of the association between deregulated ribosome biogenesis and aging

Zhang et al.^210^ compared gene expression differences by RT-PCR differential display using epithelia dissected from age-related cataracts and normal lenses. The results revealed that human age-related cataract is correlated with decreased expression of ribosomal proteins L21, L15, L13a, and L7a.

A gradual decrease in muscle mass is a common feature in older individuals. Kirby et al.^211^ showed that aged muscle failed to upregulate pre-47S ribosomal RNA (rRNA) expression, suggesting a reduction of ribosome biogenesis in aged mice.

**Fig. 7:**
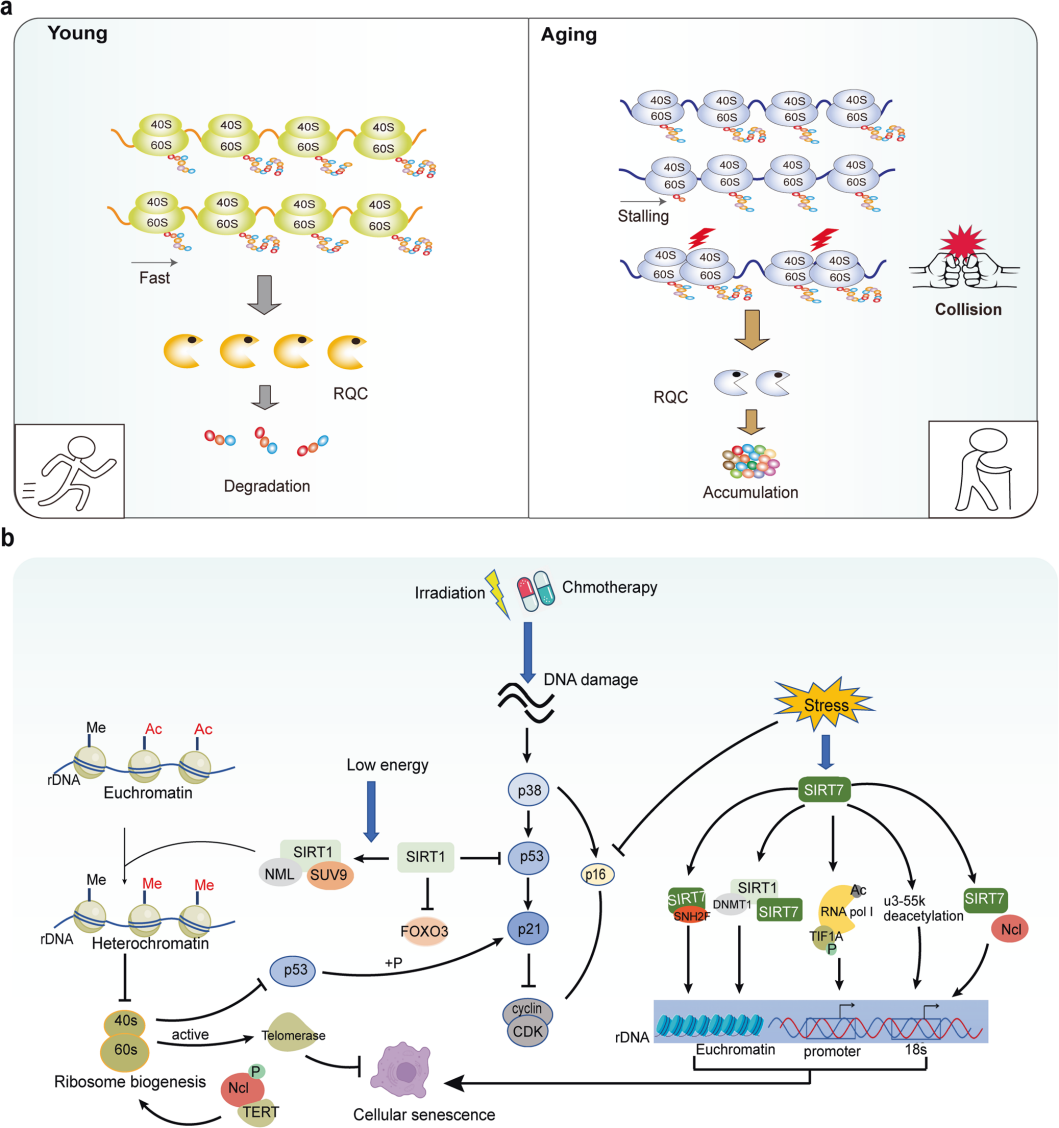
**a** Ribosome-stalling/collision/ribosome dysfunction and aging. The primary clearance pathway for ribosome collisions is the degradation of nascent peptides through ribosomal quality control (RQC). RQC decreases with age, and ribosome-stalling/collision triggers ribosome dysfunction. As a result, ribosome dysfunction leads to increased nascent polypeptides and protein aggregation. **b** Ribosomes and the signaling pathway of cellular senescence. Cellular DNA damage can be induced by radiation or chemotherapy, which activates p53-dependent stress responses and cause cellular senescence. SIRT1, a member of the longevity protein family, directly affects the activity of key proteins in the senescence pathway through deacetylating transcription factors. Moreover, SIRT1 recruits methylation enzymes to affect ribosome biogenesis by regulating chromatin remodeling, and abnormal ribosome biogenesis can directly affect p53 and telomerase activity to accelerate cellular senescence. Genomic instability of rDNA causes cellular senescence, and SIRT7, a member of the longevity protein family, affects rDNA stability by interacting with chromatin remodeling factors and RNA polymerase. Ncl and SIRT7 interact with proteins, and Ncl binding to telomeric reverse transcriptase affects ribosome biogenesis. Ac acetylation, CDK cyclin-dependent kinases, Me methylation, Ncl nucleolin, p phosphorylation, rDNA ribosomal DNA, RNA pol Ι RNA polymerase Ι

It was shown that both the translation of RP mRNA and overall mRNA translation efficiency decline with age, suggesting some defect in the process of ribosome biogenesis during aging.^212^ A recent study revealed that the ribosome of aging cells moves more slowly and periodically, causing the "stalling" of ribosome translation to increase, resulting in a collision and the accumulation of new peptides, thus aggravating ribosome dysfunction and aging.^213^ This study shed new light on the mechanism of the ribosome-stalling/collision/ribosome-dysfunction axis in age-dependent damage (Fig. 7a). The stalling of ribosomes during translation can cause protein truncation. Ribosomal Quality Control (RQC) is a mechanism to clear the stalling of ribosomes. However, the function of RQC decreased with age, leading to ribosome-stalling/collision and protein aggregation.

### The potential mechanisms contributing to deregulated ribosome biogenesis and aging

rDNA genome instability due to the accumulation of DNA damage has been implicated as a causal factor in aging.^214^ It was shown that hematopoietic stem cells build up chromosome breaks in their rDNA genes during aging.^215^ Moreover, increased rDNA instability has been found in premature aging diseases, such as Bloom and Werner syndromes.^6^

SIRT7 can stabilize SNF2H protein at the rDNA promoter. When SIRT7 is knocked out, the copy number of rDNA is reduced by 50%.^216–218^ SIRT7 also promotes RNA polymerase binding to the rDNA promoter region and coding region by catalyzing the deacetylation of RNA pol I.^219^ Moreover, SIRT7 maintains rDNA repeat stability and nucleolar integrity through the recruitment of DNA methyltransferase 1 and SIRT1.^219,220^ The nucleolus protein u3-55k is also a deacetylation substrate for SIRT7, and SIRT7/u3-55k deacetylation is required for 18s rRNA maturation and ribosome biogenesis (Fig. 7b).^221^ Radiation or chemotherapy-induced cellular senescence is mediated by p53-dependent stress responses. SIRT1 recruits methylation enzymes to affect ribosome biogenesis by regulating chromatin remodeling.

### Ribosome and neurodegenerative diseases

Studies have shown that most nervous system diseases are caused by abnormal protein synthesis, and ribosome dysfunction disrupts the homeostasis of neurons and glial cells.^222,223^

Alzheimer′s disease (AD) is a neurodegenerative disease, which is chronic and progressive, and the probability of developing dementia is as high as 60–70%.^224^ It is widely assumed that ribosome dysfunction in the cerebral cortex is one of the important pathological mechanisms of AD. Studies have found that protein levels of 5.8S and 5S rRNA are significantly reduced in early AD patients, suggesting that AD affects rRNA processing and maturation.^225^ Similarly, total rRNA levels are decreased in the brains of AD patients and are accompanied by an increase in 5S rRNA oxidation.^226^ This study indicated that the increased oxidation of 5S rRNA is associated with neurodegeneration in AD. Hernandez-Ortega et al.^227^ found that the protein levels of Ncl, and the mRNA of the upstream binding transcription factor RNA Pol I gene are decreased in late AD. In addition, alterations in the translation initiation factors eIF2α, eIF3h, eIF5, and elongation factor eEF2 have been found in AD patients.

Parkinson’s disease (PD) is an age-related neurodegenerative disorder characterized by degeneration, necrosis, and reduction of dopamine and noradrenergic neurons.^228,229^ Decreased rRNA synthesis and changes in nucleolar volume are physiological characteristics of the elderly, while aging is the major risk factor for PD. Reduced rRNA synthesis and nucleolar destruction have been reported in the dopaminergic neurons of PD mice, indicating that ribosome dysfunction may cause neuronal degeneration in PD patients.^230^ Further studies have demonstrated that rRNA synthesis and nucleolar volume are reduced in the dopaminergic neurons of PD patients, while nucleolar integrity is compromised as the disease progresses.^231^ The above results demonstrate that ribosome biogenesis is impaired in the neurodegenerative process of PD, and may be one of the factors that trigger neuronal cell death.

Accumulating evidence suggests that dysregulated ribosome biogenesis is involved in aging and neurodegenerative diseases. Future studies should focus on developing novel therapies to restore the function of ribosomes.

## Ribosomes and cancer

Multiple oncogenic signaling pathways contribute to enhanced ribosome biogenesis and protein synthesis in cancer cells. Thus, ribosome biogenesis has emerged as a potential therapeutic target. For further information on the role of ribosome biogenesis in cancer, we refer readers to other reviews.^232–235^

**Fig. 8:**
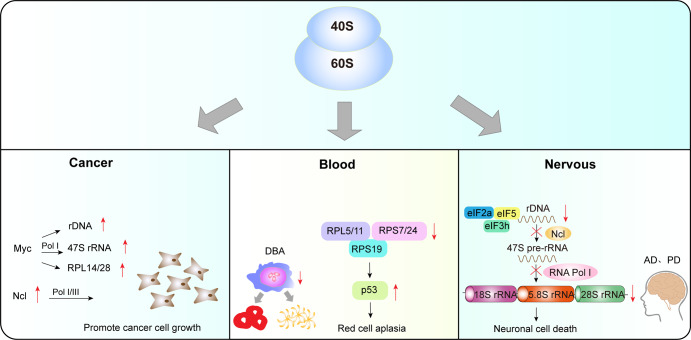
Ribosome and human diseases. Abnormally active ribosome biogenesis is critical for cancer cell growth. The oncogene Myc upregulates rDNA transcription, activates RNA pol I-mediated 47S pre-rRNA synthesis, and induces the expression of RPL14 and RPL28 to stimulate ribosome biogenesis. In addition, Ncl enables cancer cells to differentiate and grow indefinitely by increasing the RNA Pol I activity. Blood diseases are associated with abnormal expression of RPs. RPL5/11 and RPS7/24 dysfunction and mutations in RPS19 activate p53, leading to a decline or even disappearance of erythroid cells, increasing the risk of malignant transformation. Ribosome dysfunction disrupts neuronal and glial homeostasis. Abnormal rDNA transcription affects the subsequent processing of 47S pre-rRNA into mature 5.8S, 18S, and 28S rRNA, disrupting rRNA synthesis and nucleolar integrity, and ultimately inducing neuronal cell death. Myc myelocytomatosis oncogene, Ncl nucleolin, rDNA ribosomal DNA, rRNA ribosomal RNA, RNA pol Ι RNA polymerase Ι

Abnormally active ribosome biogenesis is critical for cancer cells and mediates fundamental mechanisms of cell proliferation and migration (Fig. 8).^236,237^ In cancer cells, Myc upregulates rDNA transcription by activating RNA pol I-mediated 47S pre-rRNA synthesis. Ncl promotes cancer growth by increasing the RNA Pol I activity. Blood diseases are associated with RPL5/11, RPS7/24, and RPS19 dysfunction, leading to the activation of p53. Abnormal rDNA transcription affects the processing of 47S pre-rRNA into mature 5.8S, 18S, and 28S rRNA, disrupting rRNA synthesis and nucleolar integrity, and ultimately inducing neuronal cell death. Many studies have demonstrated that RPs regulate the expression of oncogenes and cancer suppressor genes and therefore participate in the development and metastasis of cancers.^238^ The oncogene Myc acts as an active hub for the progress of cancer^239^ and increases the rate of ribosome biogenesis by directly activating rRNA synthesis in cancer cells.^39,240,241^ Meanwhile, the direct binding of Myc to rDNA activates the synthesis of 47S pre-rRNA. Of note, Myc-induced upregulation of RPL14 and RPL28 accelerates lymphoma progression. This process leads to impaired translation of the cell-cycle regulator CDK11 and genomic instability, ultimately leading to lymphatic damage.^242,243^ Therefore, one primary mechanism by which Myc induces cancer is the alterations of ribosome biogenesis.^244^

Ncl promotes rRNA transcription and pre-rRNA processing. Researchers found that Ncl combines with noncoding RNA cytoskeleton regulator RNA and then jointly participates in the development of colorectal cancer.^245^ Ncl also binds to telomerase reverse transcriptase (TERT) in the nucleoplasm and promotes its nucleolar localization, preventing cancer cells from senescence and maintaining cancer proliferation.^246^ Furthermore, Lee et al.^247^ showed that Ncl combines with astrocyte-elevated gene-1 in breast cancer to promote the growth and metastasis of breast cancer cells. Increased ribosome biogenesis in cancer cells is closely associated with continuously activated RNA Pol I/III. It promotes cell proliferation by enhancing the transcriptional activity of RNA Pol I/III and ribosome biogenesis, enabling cancer cells to differentiate and grow indefinitely.^190,248^

Ribosome biogenesis is an extremely energy-demanding process as it synthesizes the most abundant RNA and various proteins required for cell growth and proliferation. When ribosome biogenesis is impaired, cells must be able to sense the level of intracellular stress and slow down the cell cycle by altering ribosome biogenesis to avoid partial growth and improvised division. This task is accomplished by the tumor suppressor protein p53. Volarevic et al.^249^ were the first ones to describe cell cycle arrest in mouse liver cells resulting from impaired ribosome biogenesis caused by the deletion of ribosomal protein RPS6 (eS6). Since then, several studies have shown that cell cycle arrest resulting from the activation of p53 can be triggered by the interruption of ribosome biogenesis. This process has been referred to as Impaired Ribosome Biogenesis Checkpoint (IRBC).^250^

Ribosomopathies are a group of diseases caused by abnormalities of the ribosomal components. Such diseases include Diamon-Blackfan-Anemia, 5-q syndrome, SDS, X-linked DC, CHH, etc. Patients with ribosomopathies are at increased risk of cancer, bone marrow failure, and developmental abnormalities.^251^ Eμ-Myc lymphoma cells can induce short hairpin RNA expression as ribosomal protein L7a (RPL7a) or RPL11, a key component of IRBC. Only RPL7a reduction induces p53-mediated apoptosis due to the degradation of MCL-1.^252^ The evidence that IRBC responds to aberrant ribosome biogenesis to prevent oncogenesis was from the knock-in mice expressing mutant mouse double minute 2 (MDM2) C305F, which cannot complex with RPL5/RPL11/5S rRNA material combination. Crossing these mice with Eμ-Myc transgenic mice showed that c-Myc was upregulated and significantly accelerated the development of B-cell lymphomas.^253,254^ The ability of mutant MDM2 C305F to promote tumorigenesis in a c-Myc-driven cancer model is caused by the inability of the RPL5/RPL11/5S rRNA complex to upregulate p53 levels. These offer the possibility for therapeutic interventions based on IRBC-mediated p53 activation to inhibit carcinogenesis. A recent study demonstrated that IRBC could prevent cancer by reducing DNA damage and genomic instability.^255^ Impaired ribosome biogenesis caused by abnormal processing of pre-rRNA leads to nucleotide imbalances that can lead to replication stress, DNA damage, and genomic instability, which are hallmarks of cancer.^256^ Activation of IRBC prevents cancer by inducing p21-dependent G1 phase arrest, thereby preventing cells from entering the S phase in the presence of DNA damage. Many oncogenes induce nucleotide imbalance and impair ribosome biogenesis, DNA replication, and cell proliferation. Therefore, the role of IRBC in maintaining genomic stability will be crucial.^255,257^

Glutathione peroxidase 4 (GPX4) has emerged as a promising therapeutic target for cancer therapy, but some cancer cells are resistant to ferroptosis triggered by GPX4 inhibition. A recent study by Zhipeng et al.^258^ revealed the rewiring of selenoprotein hierarchies in cancer cells and identified ribosomal stall and collision during GPX4 translation as a ferroptosis vulnerability in cancer. The development of blood vessels is spatiotemporally regulated, but how this is coordinated across lineages is unclear. Katarzyna and colleagues found that the RNA helicase Ddx21, an important regulator of rRNA synthesis and ribosome biogenesis, controls Vegfc-driven developmental lymphangiogenesis by balancing endothelial ribosome biosynthesis and p53 function.^259^ This mechanism may be targeted in diseases of hyperlymphangiogenesis, such as cancer metastasis or lymphatic malformations. Fibrillin (FBL), a ribosomal biogenic factor, plays an important role in the early steps of ribosome biogenesis and is associated with poor prognosis in breast cancer when overexpressed. It was shown that a low level of FBL is a new independent marker of poor outcomes in breast cancer.^260^

Ribosome biogenesis is an essential player in cancer growth and metastasis. The development of ribosome-targeted therapy is emerging. Future works should focus on the influence of microenvironment and ribosome heterogeneity on the development of drug resistance.

## Ribosome and blood diseases

Due to the rapid renewal of bone marrow hematopoietic cells, ribosome biogenesis is involved in this process.^261,262^ Therefore, the blood system is more vulnerable to ribosome dysfunction. It has been shown that some blood diseases are associated with ribosome dysfunction.^263–265^

Diamond-Blackfan anemia (DBA) is a bone marrow failure syndrome in which the number of erythroid precursor cells in the marrow of patients is markedly decreased or even disappears, which increases the risk of malignant transformation.^266,267^ Studies have shown that a mutation of ribosomal protein S19 (RPS19) involved in the assembly of the 40S small ribosome subunit is closely associated with the pathogenesis of DBA. RPS19 is critical for hematopoietic differentiation in zebrafish, and its deletion leads to abnormal apoptosis during erythropoiesis.^268^ The overexpression of RPS19 cDNA in bone marrow cells of DBA mice reduces apoptosis, increases the formation of erythroid colonies, and attenuates bone marrow failure.^269^ The mutation of RPS19 activates p53 and causes cell senescence, and the decreased red blood cells caused by RPS19 mutation can be reversed by p53 deletion.^270^ Furthermore, it was shown that anemia is significantly alleviated in p53-deficient mice.^271^ In addition, RPS7, RPS24, RPL11, and RPL5 have also been confirmed to be closely associated with DBA.^272,273^

Shwachman-Diamond syndrome (SDS), the inherited ribosomopathy, is characterized by bone marrow failure and a high risk of myeloid malignancies at a young age. In 2003, Boocock et al.^274^ reported that the mutation of the Shwachman Bodian Diamond Syndrome (SBDS) gene is the genetic basis for developing SDS. The protein is a cofactor for elongation factor-like GTPase1 (EFL1), which removes the eukaryotic translation initiation factor 6 (eIF6) from the large ribosomal subunit, allowing the 60S subunit to form an active ribosome with the 40S ribosomal subunit.^275,276^ In particular, mutations in other genes associated with SBDS, DNAJC21, EFL1, and SRP54, all participate in the removal of eIF6 from the 60S subunit together with SBDS.^275,277^ Failure to release eIF6 can cause SDS.^278^ In addition, SBDS coprecipitated with pre-60S subunit in a sucrose gradient and bound to 28S rRNA as a component of mature 60S ribosomal subunit.^279^ Therefore, mutations in the SBDS gene lead to impaired ribosome assembly. SDS cells were also found to have aberrant expression of many genes involved in rRNA and mRNA processing and reduced expression of several ribosomal protein genes, including RPS9, RPS20, RPL6, RPL15, RPL22, RPL23, and RPL29, which are involved in cell growth and survival.^280^

X-linked dyskeratosis congenita (X-linked DC) is associated with mutations in the DKC1 gene encoding dyskerin.^281^ Dyskerin is an enzyme that catalyzes the pseudouridylation of specific uridine residues in newly synthesized rRNAs.^264^ Pseudouridines regulate ribosome and rRNA biogenesis when associated with small nucleolar RNAs (snoRNA). Cell lines from patients with X-linked DC exhibit altered regulation of snoRNAs and defects in rRNA processing.^276,282^ Furthermore, NPM1 mutations found in DC patients lead to alterations in rRNA 2’-O-methylation.^283^

Cartilage Hair Hypoplasia (CHH) is an autosomal recessive disorder caused by mutations of the RNA component of the mitochondrial RNA processing endoribonuclease (RMRP) gene.^284^ RMRP is a long noncoding RNA (lncRNA) that participates in the formation of RNase-MRP complexes and tRNA maturation. RMRP in mitochondria encodes the RNA component of RNase-MRP and is classified as snoRNAs, which are involved in several steps of ribosomal RNA synthesis.^285^ CHH fibroblasts exhibit reduced ribosome biogenesis.^276,286^

These findings imply that ribosome dysfunction is involved in the pathological process of blood diseases. Therefore, an in-depth exploration of the complex interactions between ribosome biogenesis and related signaling pathways will provide a new treatment for the diseases.

## Ribosome-targeted therapeutic strategies

Clinical trials have shown promise for ribosome-targeted therapeutic strategies, while the underlying mechanisms differ (Table 2).

**Table 2:** Clinical trials targeting cancer and ribosome biogenesis

Drugs	Diseases	Mechanism	Phase	Trial identifier
BMH-21	Cancer	BMH-21, as a first-in-class small-molecule, directly inhibits transcription elongation and DNA occupancy of RNA Pol I.	N/A	N/A
CX-5461	Cancer	CX-5461 (Pidnarulex), a synthetically-derived small molecule that selectively kills cancer cells through the binding and stabilization of G4 DNA structure	Phase 1	NCT02719977
CX-5461	Advanced solid cancer	Through the binding and stabilization of G4 DNA structure	Phase 1	NCT04890613
CX-3543 (Quarfloxin)	Advanced solid tumors, lymphoma neuroendocrine tumors, carcinoid cancer advanced solid tumors, lymphoma B-cell chronic lymphocytic leukemia	Quarfloxin is a first-in-class small-molecule targeted cancer therapeutic derived from the validated fluoroquinolone class of drugs. Quarfloxin was rationally designed to target a G-quadruplex (QPLX) DNA structure and disrupt protein-DNA interactions essential to cancer cells. The QPLX targeted by quarfloxin forms within ribosomal DNA and the QPLX is bound by the nucleolin protein	Phase 1	NCT00955786
			Phase 2	NCT00780663
			Phase 1	NCT00955292
			Phase 2	NCT00485966
Camptothecin (Topotecan and Irinotecan)	Sarcoma	Inhibits topoisomerase I and regulates early rRNA processing	Phase 3	NCT00354744
Ellipticines	Cancer	Affects the combination of SL1 with rDNA promoters, inhibits topoisomerase II and Pol I	N/A	N/A

Several new rDNA transcription inhibitors are in early clinical studies, such as CX-3543, BMH-21, and CX-5461.^287–289^, CX-3543 binds to the G4 sequence and disrupting the interaction of the rDNA G4 structure with Ncl, thereby inhibiting RNA Pol I function and inducing apoptosis in cancer cells. BMH-21 inhibits Pol I function by interacting with the rDNA backbone in GC-rich DNA sequences and also promotes the degradation of the Pol I catalytic subunit RPA194.^290^ CX-5461 decreases the binding affinity of the RNA Pol I complex to rDNA promoters and activates P53-dependent anticancer signaling. Xu et al.^291^ have shown that CX-5461 is a G-quadruplex stabilizer that kills cancer cells. DNA repair requires the BRCA and NHEJ pathways, and failure to repair leads to cell death. These data reinforce strategies for rDNA G4-targeted therapy, especially for NHEJ-deficient cancers. CX-5461 is currently in Phase I clinical trial in patients with BRCA1/2-deficient cancer (Canadian Trial, NCT02719977).

Hilton et al. reported the results of phase I trial of CX-5461 in patients with solid tumors enriched for DNA-repair deficiencies.^292^ The purpose of the trial was to determine the safety and best doses. They recruited 40 patients with solid malignant tumors who had previously failed treatment, including breast cancer (19 cases), ovarian cancer (seven cases), and pancreatic cancer (three cases). The recommended phase II dose of 475 mg/m^2^ days 1, 8, and 15 every 4 weeks were generally well tolerated. The efficacy of CX-5461 is long-lasting, and the pharmacokinetic evaluation of the trial shows that the drug can be administered once a week. In addition, the side effects and adverse reactions of CX-5461 are relatively few, and the most common adverse events are skin phototoxicity and nausea. In addition, there are a number of phase I clinical trials to evaluate the safety and tolerance of CX-5461 in the treatment of solid tumors (NCT04890613), and the efficacy of CX-5461 combined with tazopril in the treatment of castrated drug-resistant metastatic prostate cancer (NCT05425862). Although the antitumor regimen targeted by G4 DNA has been studied for many years, there are no drugs on the market, and only a handful of drugs have successfully entered clinical trials. Therefore, the performance of CX-5461 is particularly noteworthy.

In the future, we need to find an appropriate dose of ribosome biogenesis-based therapy that is efficient without causing severe side effects. Meanwhile, a more in-depth analysis of the mechanisms underlying drug resistance is needed to identify new therapeutic targets.

## Conclusions

The ribosome is an evolutionarily-conserved protein synthesis machine, and ribosome biogenesis ensures ribosome homeostasis.^293^ Ribosome biogenesis involves rDNA transcription, pre-rRNA cleavage, modification to form mature rRNA, RP synthesis and translocation into the nucleus, and rRNA assembly into large and small subunits; this is a complex and highly energy-intensive process.^176^ Therefore, different steps of ribosome biogenesis can be targeted to achieve the treatment of specific diseases. We reviewed the ribosome biogenesis and mechanisms in COVID-19, aging, the cardiovascular system, neurodegenerative disease, blood diseases, and cancer. This article aims to provide a new idea for scientists to understand how “ribosomal diseases” occur and provide a theoretical basis for developing a drug to treat such diseases.

Viruses utilize the translational machinery in host cells to achieve efficient replication. Recent researches suggest targeting ribosome biogenesis and functions is a potential new approach for controlling viral infection.^294^ CX-5461, a small-molecule drug that inhibits ribosome biogenesis, has exhibited good application prospects for malignant proliferative cancer accompanied by inhibited RNA pol I function.^175^ The SARS-CoV-2 5′UTR contains a SL1 hairpin that interact with Nsp1 to allow viral translation to proceed.^103^ Many scholars have found that the amino-terminal portion of Nsp1 is required for escape from Nsp1 inhibition.^105,295^ In order to shut down host translation, the virus maintains Nsp1 on the 40S subunit ribosome. After the viral translation is completed, the carboxy-terminal domain of Nsp1 folds back into the mRNA channel to prevent the translation of host mRNA.

We need to find a strategy to inhibit cancer precisely without affecting the function of normal cells by loading therapeutic drugs into cancer-specific targets. In addition, the implementation of precision medicine is a developing trend for the hierarchical management of different patients with different diseases, or even different patients with the same illness, based on ribosome biogenesis. It has been shown that genetic differences in ribosome occupancy can affect protein translation.^296,297^ A thorough understanding of the consequences of genetic differences among individuals is an important step toward personalized medicine. For the treatment of persons with a viral infection, the aim is to better understand the heterogeneity among individuals and determine who is most likely to benefit from a specific treatment.^298^ To achieve this goal, it is critical to develop biosensors to detect biomarkers such as viral molecules, ACE2 receptor, vitamin D levels, IgG, IgM antibodies, and C-reactive protein to help medical professionals to diagnose infection, determine the risk of infection, monitor immune response, and assess disease severity.^299^

Technological advances opened a new door to many aspects of ribosome biogenesis. Several new technologies, such as polysome profiling, ribosome-sequence, cryo-electron microscopy, and translating ribosome affinity purification, can accurately assess ribosome morphology, structure, translocation, ribosome-“frameshift”, ribosome-stalling, and ribosome-collision.^300^ For example, the abnormally expressed proteins in the plasma of patients with specific diseases can be found through ribosome-sequence technology, and the abundance of ribosomal proteins can be comprehensively monitored through Ribosome-Halo technology.^301^ These technologies not only explore the new mechanism of ribosome biogenesis and greatly expand the understanding of “ribosome diseases”, but also provide new targets for the precise diagnosis and treatment of such diseases.

## Future direction

SARS-CoV-2 hijacks the host ribosome machinery to produce its proteins. This is achieved through the interaction between the viral Nsps and the host ribosome subunits. Nsp1 binds to the 40S subunit to block the entrance for host mRNA while allowing viral mRNA to pass through and get translated. The Nsps also inhibit host mRNA nuclear export and promote its degradation. Thus, drugs or vaccines targeting viral Nsps may be a useful strategy to tackle SARS-CoV-2 infection.^302^ The big question is how many booster shots we need to prevent infection. To answer this question, it is necessary to identify biomarkers that can predict the success of a vaccine. The titer of neutralizing antibodies may not be a reliable marker since both humoral and cellular immune responses are involved in virus killing.^303,304^

Ribosome inhibitors have been among the most successful antimicrobial drugs in the past. However, the pathogens have evolved to become resistant to most antibiotics. To overcome this problem, researchers have been trying to screen new compounds from natural products of cultivatable microorganisms. However, this approach often leads to the rediscovery of an old compound. The new generation of antibiotics will rely on the discovery of new synthetic scaffolds that are designed based on high-resolution ribosome structures.^115,137^ Ribosome protection proteins such as the ATP-binding cassette subfamily F(ATP-F) are responsible for the resistance to a wide array of antibiotics.^305^ Further research on the mechanisms underlying ATP-F-mediated ribosome protection will improve our understanding of antibiotic resistance and develop more efficient therapy.
